# Supplementary material for: Discovery and application of insertion-deletion (INDEL) polymorphisms for QTL mapping of early life-history traits in Atlantic salmon
Source: BMC Genomics. 2010 Mar 8;11:156. doi: 10.1186/1471-2164-11-156 (PMC2838853; doi:10.1186/1471-2164-11-156)
Supplement: Additional file 2 — Information on developed 76 locus single-run INDEL panel in Atlantic salmon. Information on fluorescence labeling, primer concentrations, PCR pooling and links to alignments, INDEL motifs and GENESCAN (Burge and Karlin 1997) predictions of genes/exons are available in html format. [file 1471-2164-11-156-S2.ZIP › Additionalfile2/table18256.html]

Contig Alignment

```
Base	A B C D E F   
1	G . . . . .    
2	G . . . . .    
3	G . . . . .    
4	G . . . . .    
5	G . . . . .    
6	G . . . . .    
7	C . . . . .    
8	T . . . . .    
9	A . . . . .    
10	C . . . . .    
11	T . . . . .    
12	T . . . . .    
13	G . . . . .    
14	A . . . . .    
15	A . . . . .    
16	G . . . . .    
17	G . . . . .    
18	T . . . . .    
19	C . . . . .    
20	A . . . . .    
21	G . . . . .    
22	C . . . . .    
23	A . . . . .    
24	C . . . . .    
25	A . . . . .    
26	G . . . . .    
27	G . . . . .    
28	C . . . . .    
29	C . . . . .    
30	G . . . . .    
31	C . . . . .    
32	A . . . . .    
33	A . . . . .    
34	G . . . . .    
35	C . . . . .    
36	T . . . . .    
37	G . . . . .    
38	G . . . . .    
39	G . . . . .    
40	C . . . . .    
41	T . . . . .    
42	T . . . . .    
43	C . . . . .    
44	T . . . . .    
45	T . . . . .    
46	T . . . . .    
47	C . . . . .    
48	C . . . . .    
49	C . . . . .    
50	A . . . . .    
51	C . . . . .    
52	T . . . . .    
53	G . . . . .    
54	A . . . . .    
55	C . . . . .    
56	C . . . . .    
57	T . . . . .    
58	G . . . . .    
59	C . . . . .    
60	T . . . . .    
61	C . . . . .    
62	C . . . . .    
63	A . . . . .    
64	G . . . . .    
65	G . . . . .    
66	A . . . . .    
67	G . . . . .    
68	A . . . . .    
69	T . . . . .    
70	C . . . . .    
71	T . . . . .    
72	G . . . . .    
73	A . . . . .    
74	G . . . . .    
75	C . . . . .    
76	C . . . . .    
77	T . . . . .    
78	G . . . . .    
79	A . . . . .    
80	C . . . . .    
81	C . . . . .    
82	G . . . . .    
83	G . . . . .    
84	A . . . . .    
85	T . . . . .    
86	C . . . . .    
87	A . . . . .    
88	C . . . . .    
89	A . . . . .    
90	A . . . . .    
91	C . . . . .    
92	A . . . . .    
93	A . . . . .    
94	T . . . . .    
95	A . . . . .    
96	A . . . . .    
97	C . . . . .    
98	C . . . . .    
99	A . . . . .    
100	C . . . . .    
101	A . . . . .    
102	G . . . . .    
103	G . . . . .    
104	T . . . . .    
105	C . . . . .    
106	T . . . . .    
107	A . . . . .    
108	G . . . . .    
109	A . . . . .    
110	G . . . . .    
111	T . . . . .    
112	C . . . . .    
113	A . . . . .    
114	C . . . . .    
115	A . . . . .    
116	C . . . . .    
117	C . . . . .    
118	G . . . . .    
119	T . . . . .    
120	C . . . . .    
121	A . . . . .    
122	C . . . . .    
123	C . . . . .    
124	A . . . . .    
125	G . . . . .    
126	G . . . . .    
127	A . . . . .    
128	C . . . . .    
129	C . . . . .    
130	A . . . . .    
131	T . . . . .    
132	A . . . . .    
133	A . . . . .    
134	C . . . . .    
135	C . . . . .    
136	A . . . . .    
137	C . . . . .    
138	A . . . . .    
139	G . . . . .    
140	A . . . . .    
141	C . . . . .    
142	C . . . . .    
143	G . . . . .    
144	G . . . . .    
145	C . . . . .    
146	C . . . . .    
147	C . . . . .    
148	A . . . . .    
149	A . . . . .    
150	G . . . . .    
151	C . . . . .    
152	A . . . . .    
153	A . . . . .    
154	A . . . . .    
155	A . . . . .    
156	A . . . . .    
157	A . . . . .    
158	A . . . . .    
159	T . . . . .    
160	T . . . . .    
161	A . . . . .    
162	C . . . . .    
163	A . . . . .    
164	C . . . . .    
165	C . . . . .    
166	A . . . . .    
167	A . . . . .    
168	A . . A . .    
169	C . . C . .    
170	C . . C . .    
171	C . . C . .    
172	A . . A . .    
173	A . . A . .    
174	G . . G . .    
175	A . . A . .    
176	C . . C . .    
177	C . . C . .    
178	T . . T . .    
179	A . . A . .    
180	A . . A . .    
181	A . . A . .    
182	C . . C . .    
183	A . . A . .    
184	C . . C . .    
185	A . . A . .    
186	T . . T . .    
187	A . . A . .    
188	A . . A . .    
189	T . . T . .    
190	C . . C . .    
191	A . . A . .    
192	C . . C . .    
193	A . . A . .    
194	A . . A . .    
195	C . . C . .    
196	A . . A . .    
197	T . . T . .    
198	T . . T . .    
199	T . . T . .    
200	T . . T . .    
201	C . . C . .    
202	T . . T . .    
203	A . . A . .    
204	A . . A . .    
205	C . . C . .    
206	A . . A . .    
207	C . . C . .    
208	A . . A . .    
209	A . . A . .    
210	C . . C . .    
211	A . . A . .    
212	C . . C . .    
213	T . . T . .    
214	T . . T . .    
215	A . . A . .    
216	C . . C . .    
217	A . . A . .    
218	C . . C . .    
219	T . . T . .    
220	A . . A . .    
221	A . . A . .    
222	C . . C . .    
223	A . . A . .    
224	C . . C . .    
225	T . . T . .    
226	T . . T . .    
227	G . . G . .    
228	T . . T . .    
229	A . . A . .    
230	T . . T . .    
231	T . . T . .    
232	T . . T . .    
233	T . . T . .    
234	C . . C . .    
235	C . . C . .    
236	A . . A . .    
237	C . . C . .    
238	C . . C . .    
239	T . . T . .    
240	G . . G . .    
241	T . . T . .    
242	A . . A . .    
243	G . . G . .    
244	G . . G . .    
245	G . . G . .    
246	G . . G . .    
247	C . . C . .    
248	C . . C . .    
249	T . . T . .    
250	A . . A . .    
251	T . . T . .    
252	A . . A . .    
253	G . . G . .    
254	A . . A . .    
255	G . . G . .    
256	C . . C . .    
257	T . . T . .    
258	A . . A . .    
259	A . . A . .    
260	T . . T . .    
261	C . . C . .    
262	T . . T . .    
263	G . . G . .    
264	G . . G . .    
265	T . . T . .    
266	G . . G . .    
267	A . . A . .    
268	A . . A . .    
269	T . . T . .    
270	G . . G . .    
271	T . . T . .    
272	G . . G . .    
273	T . . T . .    
274	A . . A . .    
275	A . . A . .    
276	G . . G . .    
277	C . . C . .    
278	T . . T . .    
279	G . . G . .    
280	T . . T . .    
281	T . . T . .    
282	T . . T . .    
283	G . . G . .    
284	A . . A . .    
285	G . . G . .    
286	G . . G . .    
287	G . . G . .    
288	C C . C . C    
289	T T . T . T    
290	G G . G . G    
291	T T . T . T    
292	A A . A . A    
293	A A . A . A    
294	A A . A . A    
295	T T . T . T    
296	T T . T . T    
297	G G . G . G    
298	T T . T . T    
299	G G . G . G    
300	A A . A . A    
301	T T . T . T    
302	G G . G . G    
303	A A . A . A    
304	G G . G . G    
305	T T . T . T    
306	G G . G . G    
307	T T . T . T    
308	A A . A . A    
309	A A . A . A    
310	C C . C . C    
311	T T . T . T    
312	T T . T . T    
313	A A . A . A    
314	A A . A . A    
315	T T . T . T    
316	A A . A . A    
317	C C . C . C    
318	T T . T . T    
319	G G . G . G    
320	C C . C . C    
321	A A . A . A    
322	G G . G . G    
323	C C . C . C    
324	C C . C . C    
325	T T . T . T    
326	A A . A . A    
327	C C C C . C    
328	C C C C . C    
329	C C C C . C    
330	A A A A . A    
331	C C C C . C    
332	C C C C . C    
333	C C C C . C    
334	T T T T T T    
335	G G G G G G    
336	G G G G G G    
337	T T T T T T    
338	G G G G G G    
339	A A A A A A    
340	A A A A A A    
341	C C C C C C    
342	T T T T T T    
343	G G G G G G    
344	G G G G G G    
345	A A A A A A    
346	T T T T T T    
347	C C C C C C    
348	A A A A A A    
349	G G G G G G    
350	C C C C C C    
351	T T T T T T    
352	G G G G G G    
353	G G G G G G    
354	C C C C C C    
355	C C C C C C    
356	A A A A A A    
357	T T T T T T    
358	G G G G G G    
359	C C C C C C    
360	A A A A A A    
361	A A A A A A    
362	T T T T T T    
363	C C C C C C    
364	C C C C C C    
365	G G G G G G    
366	A A A A A A    
367	G G G G G G    
368	C C C C C C    
369	T T T T T T    
370	G G G G G G    
371	G G G G G G    
372	A A A A A A    
373	C C C C C C    
374	A A A A A A    
375	G G G G G G    
376	A A A A A A    
377	G G G G G G    
378	A A A A A A    
379	C C C C C C    
380	G G G G G G    
381	T T T T T T    
382	G G G G G G    
383	T T T T T T    
384	G G G G G G    
385	A A A A A A    
386	C C C C C C    
387	G G G G G G    
388	T T T T T T    
389	A A A A A A    
390	G G G G G G    
391	G G G G G G    
392	A A A A A A    
393	C C C C C C    
394	C C C C C C    
395	A A A A A A    
396	G G G G G G    
397	G G G G G G    
398	A A A A A A    
399	A A A A A A    
400	T T T T T T    
401	G G G G G G    
402	T T T T T T    
403	G G G G G G    
404	T T T T T T    
405	G G G G G G    
406	G G G G G G    
407	T T T T T T    
408	G G G G G G    
409	G G G G G G    
410	A A A A A A    
411	C C C C C C    
412	A A A A A A    
413	C C C C C C    
414	C C C C C C    
415	T T T T T T    
416	A A A A A A    
417	A A A A A A    
418	A A A A A A    
419	A A A A A A    
420	A A A A A A    
421	G G G G G G    
422	T T T T T T    
423	G G G G G G    
424	A A A A A A    
425	A A A A A A    
426	T T T T T T    
427	A A A A A A    
428	T T T T T T    
429	G G G G G G    
430	T T T T T T    
431	T T T T T T    
432	T T T T T T    
433	G G G G G G    
434	T T T T T T    
435	G G G G G G    
436	T T T T T T    
437	C C C C C C    
438	A A A A A A    
439	T T T T T T    
440	T T T T T T    
441	T T T T T T    
442	C C C C C C    
443	A A A A A A    
444	A A A A A A    
445	T T T T T T    
446	G G G G G G    
447	T T T T T T    
448	G G G G G G    
449	A A A A A A    
450	T T T T T T    
451	C C C C C C    
452	A A A A A A    
453	A A A A A A    
454	A A A A A A    
455	A A A A A A    
456	C C C C C C    
457	T T T T T T    
458	G G G G G G    
459	G G G G G G    
460	A A A A A A    
461	C C C C C C    
462	A A A A A A    
463	A A A A A A    
464	C C C C C C    
465	C C C C C C    
466	T T T T T T    
467	G G G G G G    
468	A A A A A A    
469	A A A A A A    
470	A A A A A A    
471	C C C C C C    
472	T T T T T T    
473	T T T T T T    
474	C C C C C C    
475	A A A A A A    
476	T T T T T T    
477	C C C C C C    
478	T T T T T T    
479	T T T T T T    
480	C C C C C C    
481	A A A A A A    
482	G G G G G G    
483	A A A A A A    
484	A A A A A A    
485	A A A A A A    
486	C C C C C C    
487	T T T T T T    
488	T T T T T T    
489	G G G G G G    
490	A A A A A A    
491	A A A A A A    
492	G G G G G G    
493	C C C C C C    
494	A A A A A A    
495	A A A A A A    
496	A A A A A A    
497	A A A A A A    
498	A A A A A A    
499	A A A A A A    
500	C C C C C C    
501	A A A A A A    
502	T T T T T T    
503	T T T T T T    
504	A A A A A A    
505	C C C C C C    
506	T T T T T T    
507	A A A A A A    
508	A A A A A A    
509	A A A A A A    
510	C C C C C C    
511	C C C C C C    
512	C C C C C C    
513	A A A A A A    
514	A A A A A A    
515	T T T T T T    
516	G G G G G G    
517	G G G G G G    
518	C C C C C C    
519	T T T T T T    
520	G G G G G G    
521	A A A A A A    
522	A A A A A A    
523	A A A A A A    
524	C C C C C C    
525	T T T T T T    
526	A A A A A A    
527	C C C C C C    
528	T T T T T T    
529	G G G G G G    
530	A A A A A A    
531	C C C C C C    
532	T T T T T T    
533	- G G - G G  2	 indel  
534	- T T - T T  2	 indel  
535	A A A A A A    
536	T T T T T T    
537	C C C C C C    
538	T T T T T T    
539	A A A A A A    
540	C C C C C C    
541	T T T T T T    
542	A A A A A A    
543	A A A A A A    
544	G G G G G G    
545	T T T T T T    
546	A A A A A A    
547	A A A A A A    
548	C C C C C C    
549	T T T T T T    
550	A A A A A A    
551	G G G G G G    
552	G G G G G G    
553	A A A A A A    
554	T T T T T T    
555	T T T T T T    
556	T T T T T T    
557	T T T T T T    
558	T T T T T T    
559	A A A A A A    
560	C C C C C C    
561	A A A A A A    
562	T T T T T T    
563	T T T T T T    
564	A A A A A A    
565	T T T T T T    
566	T T T T T T    
567	T T T T T T    
568	T T T T T T    
569	T T T T T T    
570	G G G G G G    
571	T T T T T T    
572	T T T T T T    
573	A A A A A A    
574	T T T T T T    
575	G G G G G G    
576	C C C C C C    
577	A A A A A A    
578	A A A A A A    
579	A A A A A A    
580	C C C C C C    
581	T T T T T T    
582	A A A A A A    
583	A A A A A A    
584	A A A A A A    
585	T T T T T T    
586	T T T T T T    
587	G G G G G G    
588	T T T T T T    
589	C C C C C C    
590	T T T T T T    
591	T T T T T T    
592	G G G G G G    
593	G G G G G G    
594	A A A A A A    
595	G G G G G G    
596	G G G G G G    
597	C C C C C C    
598	T T T T T T    
599	A A A A A A    
600	A A A A A A    
601	T T T T T T    
602	A A A A A A    
603	A A A A A A    
604	T T T T T T    
605	G G G G G G    
606	T T T T T T    
607	A A A A A A    
608	T T T T T T    
609	T T T T T T    
610	G G G G G G    
611	C C C C C C    
612	T T T T T T    
613	T T T T T T    
614	A A A A A A    
615	C C C C C C    
616	T T T T T T    
617	C C C C C C    
618	T T T T T T    
619	T T T T T T    
620	A A A A A A    
621	T T T T T T    
622	T T T T T T    
623	G G G G G G    
624	T T T T T T    
625	A A A A A A    
626	G G G G G G    
627	T T T T T T    
628	A A A A A A    
629	G G G G G G    
630	T T T T T T    
631	T T T T T T    
632	C C C C C C    
633	C C C C C C    
634	T T T T T T    
635	T T T T T T    
636	T T T T T T    
637	T T T T T T    
638	T T T T T T    
639	A A A A A A    
640	T T T T T T    
641	A A A A A A    
642	T T T T T T    
643	A A A A A A    
644	T T T T T T    
645	A A A A A A    
646	G G G G G G    
647	T T T T T T    
648	C C C C C C    
649	A A A A A A    
650	G G G G G G    
651	C C C C C C    
652	T T T T T T    
653	G G G G G G    
654	A A A A A A    
655	A A A A A A    
656	A A A A A A    
657	T T T T T T    
658	T T T T T T    
659	C C C C C C    
660	T T T T T T    
661	A A A A A A    
662	G G G G G G    
663	T T T T T T    
664	G G G G G G    
665	C C C C C C    
666	T T T T T T    
667	T T T T T T    
668	A A A A A A    
669	A A A A A A    
670	T T T T T T    
671	T T T T T T    
672	T T T T T T    
673	T T T T T T    
674	C C C C C C    
675	C C C C C C    
676	C C C C C C    
677	C C C C C C    
678	T T T T T T    
679	T T T T T T    
680	G G G G G G    
681	A A A A A A    
682	T T T T T T    
683	G G G G G G    
684	C C C C C C    
685	A A A A A A    
686	A A A A A A    
687	A A A A A A    
688	T T T T T T    
689	G G G G G G    
690	T T T T T T    
691	T T T T T T    
692	C C C C C C    
693	A A A A A A    
694	A A A A A A    
695	G G G G G G    
696	G G G G G G    
697	G G N G G G    
698	G G G G G G    
699	G G G G G G    
700	C C C C C C    
701	T T T T T T    
702	T T T T T T    
703	G G G G G G    
704	T T T T T T    
705	T T T T T T    
706	A A A A A A    
707	A A A A A A    
708	A A A A A A    
709	G G G G G G    
710	T T T T T T    
711	G G G G G G    
712	C C C C C C    
713	A A A A A A    
714	T T T T T T    
715	G G G G G G    
716	T T T T T T    
717	T T T T T T    
718	G G G G G G    
719	T T T T T T    
720	A A A A A A    
721	A A A A A A    
722	T T T T T T    
723	T T T T T T    
724	T T T T T T    
725	T T T T T T    
726	T T T T T T    
727	T T T T T T    
728	T T T T T T    
729	G G G G G G    
730	C C C C C C    
731	T T T T T T    
732	T T T T T T    
733	C C C C C C    
734	G G G G G G    
735	G G G G G G    
736	C C C C C C    
737	C C C C C C    
738	A A A A A A    
739	G G G G G G    
740	T T T T T T    
741	C C C C C C    
742	T T T T T T    
743	T T T T T T    
744	T T T T T T    
745	T T T T T T    
746	A A A A A A    
747	T T T T T T    
748	C C C C C C    
749	A C C A C C  2	 transversion  
750	T T T T T T    
751	C C C C C C    
752	T T T T T T    
753	T T T T T T    
754	T T T T T T    
755	A A A A A A    
756	A A A A A A    
757	C C C C C C    
758	A A A A A A    
759	A A A A A A    
760	A A A A A A    
761	A A A A A A    
762	C C C C C C    
763	A A A A A A    
764	G G G G G G    
765	T T T T T T    
766	G G G G G G    
767	T T T T T T    
768	G G G G G G    
769	C C C C C C    
770	T T T T T T    
771	G G G G G G    
772	T T T T T T    
773	G G G G G G    
774	T T T T T T    
775	A A A A A A    
776	T T T T T T    
777	T T T T T T    
778	T T T T T T    
779	T T G G G G  2	 transversion  
780	. . T T T T    
781	. . T T T T    
782	. . G G G G    
783	. . T T T T    
784	. . A A A A    
785	. . T T T T    
786	. . T T T T    
787	. . T T T T    
788	. . A A A A    
789	. . T T T T    
790	. . A A A A    
791	. . T T T T    
792	. . A A A A    
793	. . G G G G    
794	. . A A A A    
795	. . G G G G    
796	. . C C C C    
797	. . A A A A    
798	. . A A A A    
799	. . T T T T    
800	. . A A A A    
801	. . G G G G    
802	. . A A A A    
803	. . C C C C    
804	. . A A A A    
805	. . A A A A    
806	. . T T T T    
807	. . . G G G    
808	. . . T T T    
809	. . . A A A    
810	. . . A A A    
811	. . . G G G    
812	. . . C C C    
813	. . . C C C    
814	. . . T T T    
815	. . . G G G    
816	. . . G G G    
817	. . . C C C    
818	. . . T T T    
819	. . . G G G    
820	. . . A A A    
821	. . . C C C    
822	. . . A A A    
823	. . . G G G    
824	. . . A A A    
825	. . . G G G    
826	. . . A A A    
827	. . . C C C    
828	. . . C C C    
829	. . . C C C    
830	. . . A A A    
831	. . . C C C    
832	. . . A A A    
833	. . . C C C    
834	. . . A A A    
835	. . . C C C    
836	. . . T T T    
837	. . . T T T    
838	. . . T T T    
839	. . . T T T    
840	. . . G G G    
841	. . . C C C    
842	. . . C C C    
843	. . . A A A    
844	. . . G G G    
845	. . . A A A    
846	. . . C C C    
847	. . . A A A    
848	. . . A A A    
849	. . . A A A    
850	. . . T T T    
851	. . . G G G    
852	. . . T T T    
853	. . . A A A    
854	. . . G G G    
855	. . . G G G    
856	. . . C C C    
857	. . . C C C    
858	. . . T T T    
859	. . . A A A    
860	. . . T T T    
861	. . . C C C    
862	. . . A A A    
863	. . . T T T    
864	. . . T T T    
865	. . . G G G    
866	. . . A A A    
867	. . . T T T    
868	. . . G G G    
869	. . . C C C    
870	. . . A A A    
871	. . . A A A    
872	. . . T T T    
873	. . . G G G    
874	. . . T T T    
875	. . . T T T    
876	. . . G G G    
877	. . . T T T    
878	. . . A A A    
879	. . . A A A    
880	. . . C C C    
881	. . . A A A    
882	. . . T T T    
883	. . . T T T    
884	. . . A A A    
885	. . . T T T    
886	. . . G G G    
887	. . . C C C    
888	. . . T T T    
889	. . . A A A    
890	. . . C C C    
891	. . . A A A    
892	. . . G G G    
893	. . . T T T    
894	. . . C C C    
895	. . . A A A    
896	. . . T T T    
897	. . . T T T    
898	. . . T T T    
899	. . . G G G    
900	. . . T T T    
901	. . . T T T    
902	. . . A A A    
903	. . . T T T    
904	. . . T T T    
905	. . . A A A    
906	. . . A A A    
907	. . . T T T    
908	. . . T T T    
909	. . . T T T    
910	. . . A A A    
911	. . . C C C    
912	. . . T T T    
913	. . . A A A    
914	. . . C C C    
915	. . . T T T    
916	. . . G G G    
917	. . . A A A    
918	. . . A A A    
919	. . . A A A    
920	. . . T T T    
921	. . . A A A    
922	. . . C C C    
923	. . . T T T    
924	. . . A A A    
925	. . . A A A    
926	. . . C C C    
927	. . . A A A    
928	. . . G G G    
929	. . . G G G    
930	. . . G G G    
931	. . . G G G    
932	. . . A A A    
933	. . . G G G    
934	. . . T T T    
935	. . . G G G    
936	. . . C C C    
937	. . . A A A    
938	. . . G G G    
939	. . . G G G    
940	. . . C C C    
941	. . . T T T    
942	. . . T T T    
943	. . . T T T    
944	. . . T T T    
945	. . . C C C    
946	. . . C C C    
947	. . . C C C    
948	. . . A A A    
949	. . . T T T    
950	. . . T T T    
951	. . . T T T    
952	. . . A A A    
953	. . . T T T    
954	. . . T T T    
955	. . . G G G    
956	. . . T T T    
957	. . . C C C    
958	. . . T T T    
959	. . . T T T    
960	. . . T T T    
961	. . . C C C    
962	. . . T T T    
963	. . . T T T    
964	. . . G G G    
965	. . . A A A    
966	. . . T T T    
967	. . . A A A    
968	. . . T T T    
969	. . . G G G    
970	. . . C C C    
971	. . . A A A    
972	. . . T T T    
973	. . . C C C    
974	. . . C C C    
975	. . . T T T    
976	. . . C C C    
977	. . . T T T    
978	. . . G G G    
979	. . . T T T    
980	. . . G G G    
981	. . . A A A    
982	. . . C C C    
983	. . . A A A    
984	. . . C C C    
985	. . . C C C    
986	. . . A A A    
987	. . . T T T    
988	. . . C C C    
989	. . . A A A    
990	. . . T T T    
991	. . . A A A    
992	. . . G G G    
993	. . . T T T    
994	. . . G G G    
995	. . . G G G    
996	. . . T T T    
997	. . . G G G    
998	. . . A A A    
999	. . . A A A    
1000	. . . A A A    
1001	. . . T T T    
1002	. . . C C C    
1003	. . . A A A    
1004	. . . A A A    
1005	. . . T T T    
1006	. . . T T T    
1007	. . . C C C    
1008	. . . A A A    
1009	. . . C C C    
1010	. . . A A A    
1011	. . . A A A    
1012	. . . T T T    
1013	. . . T T T    
1014	. . . C C C    
1015	. . . G G G    
1016	. . . A A A    
1017	. . . A A A    
1018	. . . A A A    
1019	. . . T T T    
1020	. . . A A A    
1021	. . . A A A    
1022	. . . A A A    
1023	. . . G G G    
1024	. . . T T T    
1025	. . . C C C    
1026	. . . C C C    
1027	. . . T T T    
1028	. . . C C C    
1029	. . . T T T    
1030	. . . T T T    
1031	. . . T T T    
1032	. . . T T T    
1033	. . . T T T    
1034	. . . T T T    
1035	. . . A A A    
1036	. . . A A A    
1037	. . . T T T    
1038	. . . C C C    
1039	. . . A A .    
1040	. . . G G .    
1041	. . . T T .
```
